# Supplementary material for: HU Searches and Binds Specific DNA via a Multistep Process Combining Weak Electrostatic Binding, Protein Reorientation, and DNA Flexibility
Source: JACS Au. 2025 Oct 14;5(10):4870–8. doi: 10.1021/jacsau.5c00800 (PMC12569673; doi:10.1021/jacsau.5c00800)
Supplement: Supplementary file 1 [file au5c00800_si_001.pdf]

Supplementary Material:

HU searches and binds specific DNA via a multi-step process combining weak electrostatic binding, protein reorientation and DNA flexibility

Elliot W. Chan[1,2], Mark C. Leake[1,3,4] Agnes Noy[1,4] *

[1] School of Physics, Engineering and Technology, University of York, York, UK
[2] School of Chemistry, University of Bristol, Bristol, UK
[3] Department of Biology, University of York, York, UK
[4] York Biomedical Research Institute, University of York, York, UK

Supplementary Figures

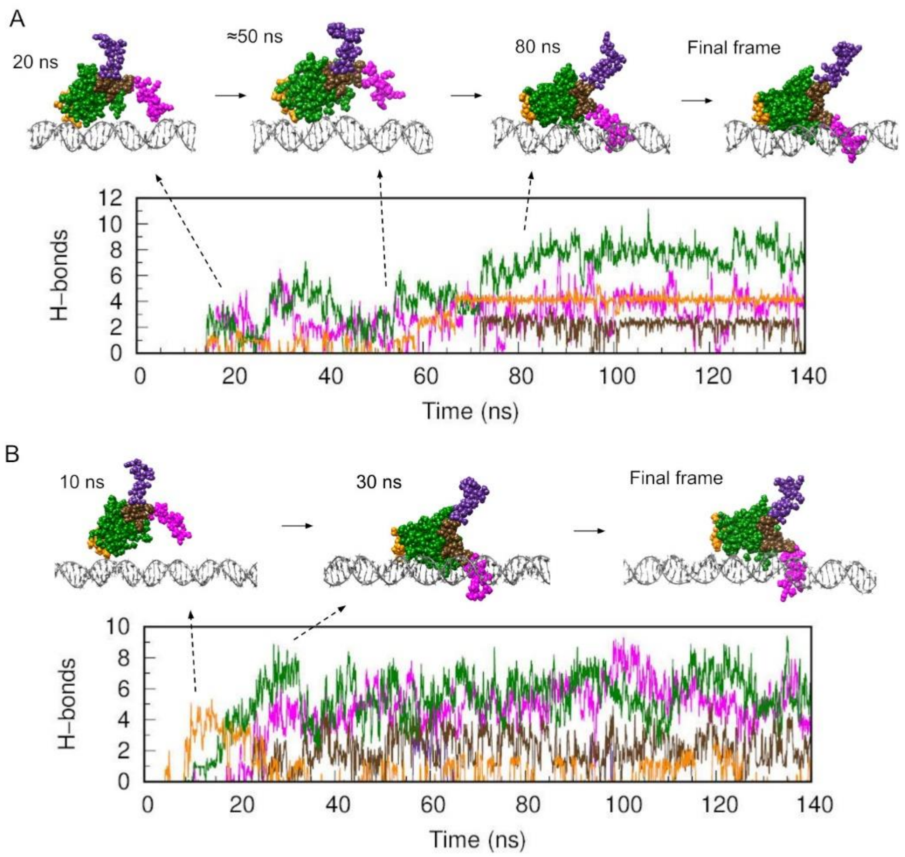

Figure S1: Time evolution of hydrogen bonds formation between DNA and various HU regions (color code as in Figure 1) together with representative frames from replicas 6 (A) and 7 (B) from the ten calculated using a 150-bp DNA solvated in a rectangular solvation box and initiated with HU 3 nm away.

Supplementary Movies

Movie S1. Movie of the preliminary simulation (replica 0), where HU hops over a 60-bp DNA.

Movie S2. Movie of the first replica of the ten performed with a 150-bp DNA, where HU jumps from the DNA of the central solvation box solvated to its periodic boundary copy at the top.

Movie S3. Movie of the second replica of the ten performed with a 150-bp DNA, where HU oscillates back and forth over DNA, until the protein binds via the lateral site, as in the search mode.

Movie S4. Movie of the third replica of the ten performed with a 150-bp DNA, where HU initially binds the DNA with its bottom and then rolls over it, until the protein binds via the lateral site, as in the search mode.

Movie S5. Movie of the fourth replica of the ten performed with a 150-bp DNA, where HU initially binds the DNA with its bottom and then rolls over it, until the protein binds via the lateral site, as in the search mode.

Movie S6. Movie of the fifth replica of the ten performed with a 150-bp DNA, where HU binds directly DNA via the lateral site, as in the search mode.

Movie S7. Movie of the simulation started with HU bound to B-DNA as in pdb 4YEW, remaining in the search mode.

Movie S8. Movie of the simulation started with HU bound to damaged DNA as in pdb 4YEW, which transitions from the search mode or non-specific binding to the recognition mode or specific binding as observed in pdb 1PT8.
